# The Augmented Lagrange Multipliers Method for Matrix Completion from Corrupted Samplings with Application to Mixed Gaussian-Impulse Noise Removal

**DOI:** 10.1371/journal.pone.0108125

**Published:** 2014-09-23

**Authors:** Fan Meng, Xiaomei Yang, Chenghu Zhou

**Affiliations:** 1 Institute of Geographic Sciences and Natural Resources Research, Chinese Academy of Sciences, Beijing, China; 2 University of Chinese Academy of Sciences, Beijing, China; Institute of Psychology, Chinese Academy of Sciences, China

## Abstract

This paper studies the problem of the restoration of images corrupted by mixed Gaussian-impulse noise. In recent years, low-rank matrix reconstruction has become a research hotspot in many scientific and engineering domains such as machine learning, image processing, computer vision and bioinformatics, which mainly involves the problem of matrix completion and robust principal component analysis, namely recovering a low-rank matrix from an incomplete but accurate sampling subset of its entries and from an observed data matrix with an unknown fraction of its entries being arbitrarily corrupted, respectively. Inspired by these ideas, we consider the problem of recovering a low-rank matrix from an incomplete sampling subset of its entries with an unknown fraction of the samplings contaminated by arbitrary errors, which is defined as the problem of matrix completion from corrupted samplings and modeled as a convex optimization problem that minimizes a combination of the nuclear norm and the 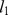-norm in this paper. Meanwhile, we put forward a novel and effective algorithm called augmented Lagrange multipliers to exactly solve the problem. For mixed Gaussian-impulse noise removal, we regard it as the problem of matrix completion from corrupted samplings, and restore the noisy image following an impulse-detecting procedure. Compared with some existing methods for mixed noise removal, the recovery quality performance of our method is dominant if images possess low-rank features such as geometrically regular textures and similar structured contents; especially when the density of impulse noise is relatively high and the variance of Gaussian noise is small, our method can outperform the traditional methods significantly not only in the simultaneous removal of Gaussian noise and impulse noise, and the restoration ability for a low-rank image matrix, but also in the preservation of textures and details in the image.

## Introduction

Image denoising is highly demanded in the field of image processing, since noise is usually inevitable during the process of image acquisition and transmission, which significantly degrades the visual quality and makes subsequent high-level image analysis and understanding very difficult. There exist two types of most common and extensively studied noise: additive Gaussian noise and impulse noise. Additive Gaussian noise is usually generated during image acquisition and characterized by adding each image pixel a random value from the Gaussian distribution with zero mean and standard deviation 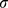, while impulse noise is very different in nature from Gaussian noise. Impulse noise can often be introduced in both image acquisition and transmission process by malfunctioning pixels in camera sensors, faulty memory locations in hardware, or transmission in a noisy channel, which mainly includes two models, namely salt-pepper noise and random-valued impulse noise [1]. For the former model, each gray value is replaced with a given probability 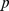 by the extreme value 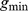 or 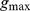, leaving remaining pixels unchanged, where 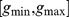 denotes the dynamic range of an image and 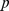 determines the level of the salt-pepper noise; as for the latter case, the intensity values of contaminated pixels are taken place by random values identically and uniformly distributed in 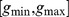. In this paper, we will focus on the first model of impulse noise. A fundamental target of image denoising is to effectively remove noises from an image while preserving image details and textures.

For Gaussian noise removal, the non-local means (NL-means) proposed by Buades A. et al. [2] is quite an efficient method in suppressing Gaussian noise while keeping details and structures in the image intact, which better exploits the redundancy of natural images and calculates the weighted average of all the pixels in the image based on similarity of neighborhoods, to achieve a satisfying filtering result. Inspired by nonlocal concept, many more nonlocal methods such as BM3D [3] and K-SVD [4] have been introduced and get state-of-the-art denoising performance for Gaussian noise. In addition, some filters based on wavelet transform [5] and partial differential equations (PDE) [6], [7] including nonlinear total variation (TV) are also powerful tools for it. The traditional approaches for impulse noise removal operate locally and nonlinearly on images. Among them, let us mention the classical median filter and its variants like adaptive median filter (AMF), progressive switching median filter (PSMF), and direction weighting median filter (DWMF). This kind of nonlinear filters can remove impulse noise effectively but cannot preserve image details well. In order to better preserve edge structures in images, variational approaches have been developed. In [8], a data-fidelity term of 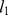-norm was first introduced to achieve a significant improvement to remove outliers like impulse noise. Some authors [9], [10], [11] proposed two-phase schemes to better preserve image details: the main idea is to detect the location of noisy pixels corrupted by impulse noise using median-like filters, followed by some variational methods to estimate the gray values for those contaminated pixels.

However, most existing image denoising methods can only deal with a single type of noise, which violates the fact that most of the image noise we encounter in real world can normally be represented by a mixture of Gaussian and impulse noise. Effectively eliminating mixed noise is difficult due to the distinct characteristics of both types of degradation processes. It should be noted that the two-phase approaches [12]–[16] also show exuberant vitality for mixed noise removal, namely detecting or estimating the outliers before proceeding with the restoration phase. Garnett et al. [12] introduced a universal noise removal algorithm that first detects the impulse corrupted pixels, estimates its local statistics and incorporates them into the bilateral filter, resulting in the trilateral filter. In [13] somewhat similar ideas were used to establish a similarity principle which in turn supplies a simple mathematical justification for the nonlocal means filter in removing Gaussian noises. This is also the case of [15] where outliers are first detected by a median-like filter and then a K-SVD dictionary learning is performed on impulse free pixels to solve a 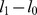 minimization problem, and the case of [16] where a median-type filter is used to remove impulse noise first and then NL-means based method is applied to remove the remaining Gaussian noise. Liu et al. [18] proposed a general weighted 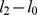 norms energy minimization model to remove mixed noise, which was built upon maximum likelihood estimation framework and sparse representations over a trained dictionary. While in [19], weighted encoding with sparse nonlocal regularization was put forward for mixed noise removal, where there was not an explicit step of impulse pixel detection, and soft impulse pixel detection via weighted encoding was used to deal with impulse and Gaussian noise simultaneously instead. See [17], [34], [35] for more approaches to mixed noise removal. Although these denoising methods above were proposed specially for mixed Gaussian-impulse removal and indeed can alleviate the impact on image visual effect brought by mixed noises to some extent, most of them will erase details, and cannot better preserve regularly geometrical textures and fine structures.

In recent years, low-rank matrix reconstruction has become a research hotspot in many scientific and engineering domains, with myriad applications ranging from web search to machine learning to computer vision and image analysis, which mainly involves the problem of matrix completion (MC) [20], [21], [26], [27] and robust principal component analysis (RPCA) [22]–[25], [28], namely recovering a low-rank matrix from an incomplete but accurate sampling subset of its entries and from an observed data matrix with an unknown fraction of its entries being arbitrarily corrupted, respectively. Luckily, there usually exist many regularly geometrical textures and fine structures in both natural image and Remote Sensing (RS) image, due to the self-similarity and redundancy of image, which makes the grayscale matrix of the image possess low-rank features. In light of this, we propose a novel denoising framework for better preserving details and low-rank features in images while removing mixed Gaussian-impulse noise, based on the theory of low-rank matrix reconstruction.

In this paper, we consider the problem of recovering a low-rank matrix from an incomplete sampling subset of its entries with an unknown fraction of the samplings contaminated by arbitrary errors, which is defined as the problem of matrix completion from corrupted samplings (MCCS) and modeled as a convex optimization problem that minimizes a combination of the nuclear norm and the 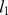-norm. To exactly solve the problem, we introduce an effective algorithm called augmented Lagrange multipliers (ALM). By detecting impulse noises in a noisy image and treating the impulse free entries of the image matrix as available samplings first, and then regarding the Gaussian noises underlying the samplings as arbitrary errors, we can exploit the proposed algorithm to remove mixed noises and to recover the image matrix with low-rank or approximately low-rank features. To sum up, the main contributions of the paper include modeling the problem of low-rank matrix recovery from incomplete and corrupted samplings of its entries, solving the convex optimization problem via the proposed ALM algorithm and creatively applying it to mixed Gaussian-impulse noise removal.

The rest of the paper is organized as follows. We start in Section 2 by giving some introductions about the theory of low-rank matrix recovery including the problem of MC and Robust PCA. We put forward the ALM algorithm for the problem of MCCS and describe some implementation details and parameter settings for our methods in Section 3, in which we also analyze its powerful performance for exact recovery of low-rank matrices with erasures and errors. In Section 4, we will present in details our full denoising schemes for the impulse detector and mixed Gaussian-impulse noise removal. Experiments and comparisons with recent approaches are demonstrated in Section 5. Finally, we conclude this paper in Section 6.

## Low-Rank Matrix Reconstruction

Low-rank matrix plays a central role in large-scale data analysis and dimensionality reduction, since low-rank structure is usually used either to approximate a general matrix, or to recover corrupted or missing data [25]. From the mathematical viewpoint, these practical problems come down to the theory of low-rank matrix reconstruction, which mainly involves the problem of MC and Robust PCA at present.

### 1 Matrix completion

To describe the problem of MC, suppose to simplify that the unknown matrix 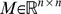 is square, and that one has 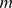 sampled entries 
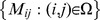
 where 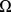 is a random subset of cardinality 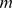. The recovery of complete matrix 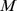 from these incomplete samplings of its entries is the MC problem.

The issue is of course that this problem is extraordinarily ill-posed because, with fewer samples than entries, there are infinitely many completions. Therefore, it is apparently impossible to identify which of these candidate solutions is indeed the “correct” one without some additional information. In many instances, however, the matrix we wish to recover has low rank or approximately low rank, which may change the property of the problem, and make the search for solutions feasible since the lowest-rank solution now tends to be the right one.

Candes and Recht [20] showed that matrix completion is not as ill-posed as once thought. Indeed, they proved that most low-rank matrices can be recovered exactly from most sets of sampled entries even though these sets have surprisingly small cardinality, and more importantly, they proved that this can be done by solving a simple convex optimization problem
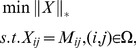
(1)provided that the number of samples obeys
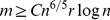
(2)for some positive numerical constant 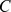, where 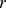 is the rank of matrix 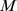. In (1), the notation 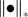 denotes the nuclear norm of a matrix, which is the sum of its singular values. The optimization problem (1) is convex and can be recast as a semidefinite program. In some sense, this is the tightest convex relaxation of the NP-hard rank minimization problem
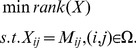
(3)


Another interpretation of Candes and Recht's result is that under suitable conditions, the rank minimization program (3) and the convex program (1) are formally equivalent in the sense that they have exactly the same unique solution. The state-of-the-art algorithms to solve the MC problem include the accelerated proximal gradient (APG) approach and the singular value thresholding (SVT) method [21].

### 2 Robust principal component analysis

Principal component analysis (PCA), as a popular tool for high-dimensional data processing, analysis, compression and visualization, has wide applications in scientific and engineering fields. It assumes that the given high-dimensional data lie near a much lower-dimensional linear subspace. To large extent, the goal of PCA is to efficiently and accurately estimate this low-dimensional subspace.

Suppose that the given data are arranged as the columns of a large matrix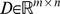. The mathematical model for estimating the low-dimensional subspace is to find a low-rank matrix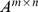, such that the discrepancy between 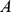 and 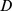 is minimized, leading to the following constrained optimization: 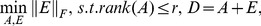
(4)where 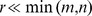 is the target dimension of the subspace and 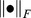 is the Frobenius norm, which corresponds to assuming that the data are corrupted by i.i.d. Gaussian noise. This problem can be conveniently solved by first computing the Singular Value Decomposition (SVD) of 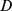, and then projecting the columns of 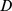 onto the subspace spanned by the 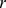 principal left singular vectors of 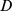.

As PCA gives the optimal estimate when the corruption is caused by additive i.i.d. Gaussian noise, it works well in practice as long as the magnitude of noise is small. However, it breaks down under large corruption, even if that corruption only affects very few of the observations. Therefore, it is necessary to study whether a low-rank matrix 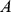 can still be efficiently and accurately recovered from a corrupted data matrix 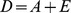, where some entries of the additive error matrix 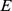 may be arbitrarily large.

Recently, Wright et al. [23] have shown that under rather broad conditions the answer is affirmative: provided the error matrix 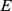 is sufficiently sparse, one can exactly recover the low-rank matrix 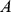 from 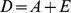 by solving the following convex optimization problem:
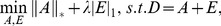
(5)where 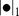 denotes the sum of the absolute values of matrix entries, and 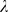 is a positive weighting parameter. Due to the ability to exactly recover underlying low-rank structure in the data, even in the presence of large errors or outliers, this optimization is referred to as Robust PCA. So far, RPCA has been successfully introduced into several applications such as background modeling and removing shadows and specularities from face images. Ganesh et al. [31] proposed two new algorithms for solving the problem (5), which are in some sense complementary to each other. The first one is an accelerated proximal gradient algorithm applied to the primal, which is a direct application of the FISTA framework introduced by [30], coupled with a fast continuation technique; the other one is a gradient-ascent approach applied to the dual of the problem (5). Recently, the augmented Lagrange multiplier method was introduced by Lin et al. for exact recovery of corrupted low-rank matrices, which shows rather promising performance when dealing with the problem (5).

## Problem of MCCS and the ALM Algorithm

### 1 MCCS and its optimization model

Since the MC problem is closely connected to the RPCA problem, we may formulate the MC problem in the same way as RPCA
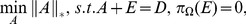
(6)where 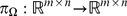 is a linear operator that keeps the entries in 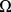 unchanged and sets those outside 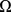 (i.e., in 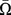) zeros. As 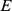 will compensate for the unknown entries of 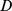, the unknown entries of 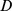 are simply set as zeros. Then the partial augmented Lagrangian function of (6) is
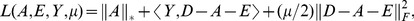
(7)where 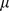 is a positive scalar. For updating 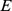, the constraint 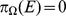 should be enforced when minimizing 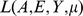.

The problem of restoring matrix from corrupted entries (i.e., RPCA) is less studied than the simpler MC problem when the available entries are not corrupted. However, in many practical applications, there usually exists the case when missing entries and corrupted entries simultaneously concur in an observed data matrix, which urgently drives researchers to pay more and more attention to the problem of recovering low-rank matrix from its observed data matrix with erasures and errors.

For observed matrix 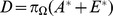 where the ordered pair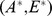 denotes the true solutions to low-rank matrix and sparse error matrix respectively, suppose that one can only know the entries of 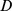 on subset 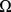 (the set of indices of known entries), and that one aims to recover low-rank matrix 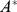 from the observed matrix 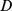 with erasures and errors. In this paper, we define the problem as matrix completion from corrupted samplings or robust matrix completion, which is the problem of restoring low-rank matrix from incomplete and corrupted samplings.

Similarly, as the entries of matrix 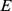 on set 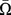 will compensate for the unknown entries of 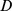 and the entries of 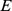 on set 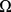 are sparse, we can split 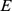 into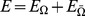, where 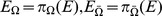 and 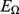 denotes sparse error components for the known entries. Now we can formulate the problem of MCCS as follows:
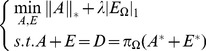
(8)


To recover the low-rank matrix for the MCCS problem (8), we propose a novel and effective algorithm based on the ALM method, whose objective function is as follows:
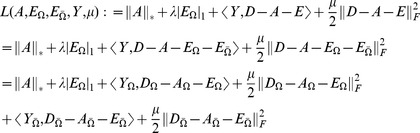
(9)


In (9), as 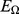 and 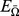 are complementary to each other, we can ignore one when solving another one, and finally combine the two to obtain 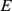. Unlike the technique presented in [29], we separate 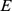 into two parts in the objective function, which leads to the splitting of 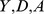 afterwards, and aims to achieve subsection optimization.

### 2 The proposed ALM algorithm and parameter settings

The ALM algorithm for the Robust MC problem proposed in the paper is described in Algorithm 1. It is apparent that computing the full SVD for the Robust MC problem is unnecessary, so we only need those singular values that are larger than a particular thresholding and their corresponding singular vectors. Firstly, we should predict the dimension of principal singular space, and in the paper, we exploit the following prediction rule:
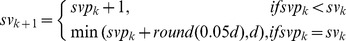
(10)where 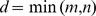, 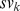 is the predicted dimension and 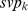 denotes the number of singular values in the 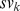 singular values that are larger than 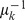, and 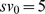. In the paper, we choose 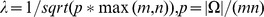, and set 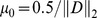. The following conditions are chosen as the stopping criterion:
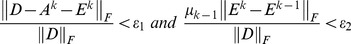
(11)


For convenience, we introduce the following soft-thresholding (shrinkage) operator:
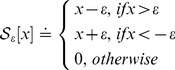
(12)where 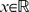 and 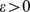. This operator can be extended to vectors and matrices by applying it element-wise. In Algorithm 1, 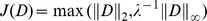, where 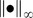 is the maximum absolute value of the matrix entries, and 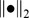 denotes the maximum singular value of a matrix.
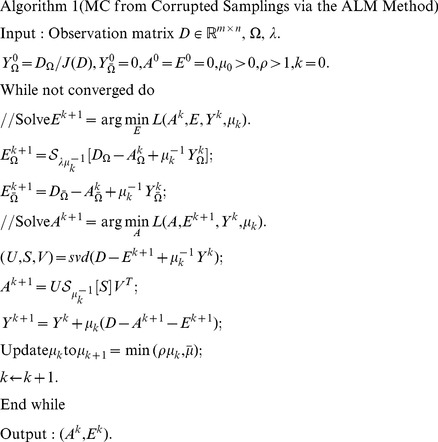



### 3 Performance analysis of the proposed algorithm

In this subsection, we first demonstrate the recovery accuracy of the proposed ALM algorithm by simulation experiments; and then for clarifying how the erasure rate (the fraction of the unknown entries in observed matrix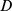, i.e.,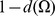,where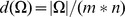) and the error probability (the fraction of randomly corrupted entries in the known entries available for restoration, i.e.,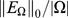,where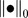 denotes the sparsity of a matrix) affect the performance of our method, we carry out the phase transition analysis for the proposed algorithm. We generate the rank-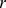 matrix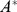 as a product 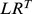, where 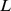 and 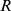 are independent 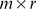 matrices whose elements are i.i.d. Gaussian random variables with zero mean and unit variance, and set a fraction of entries chosen randomly in 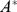 as zeros; and then, treat a fraction of non-zero entries also chosen randomly in 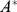 as corrupted entries after adding arbitrarily large errors 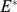 to them; finally, we get the observed data matrix 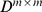 with erasures valued zeros.

We exploit different data matrices and various combinations 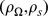 to do the experiments, where 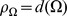 and 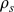 stands for the error probability, and the reconstruction results are presented in Table 1. We observe that the proposed algorithm can accurately recover the low-rank matrices and sparse errors even when erasure rate reaches 30% and 20% of the available entries are corrupted.

**Table 1:** Recovery results of low-rank matrices with different 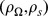 using the proposed ALM.

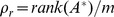	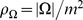	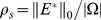	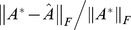	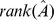	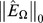
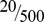	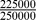	0.1	1.20e-6	20	22500
		0.2	2.47e-6	20	45000
	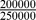	0.1	1.38e-6	20	20000
		0.2	2.22e-6	20	39999
	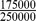	0.1	1.27e-6	20	17500
		0.2	2.54e-6	20	35109
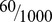	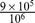	0.1	6.20e-7	60	90000
		0.2	4e-4	60	181894
	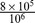	0.1	6.82e-7	60	80000
		0.2	2e-4	60	163523
	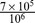	0.1	4.84e-7	60	70233
		0.2	7.47e-4	60	154281

**Figure 1:**
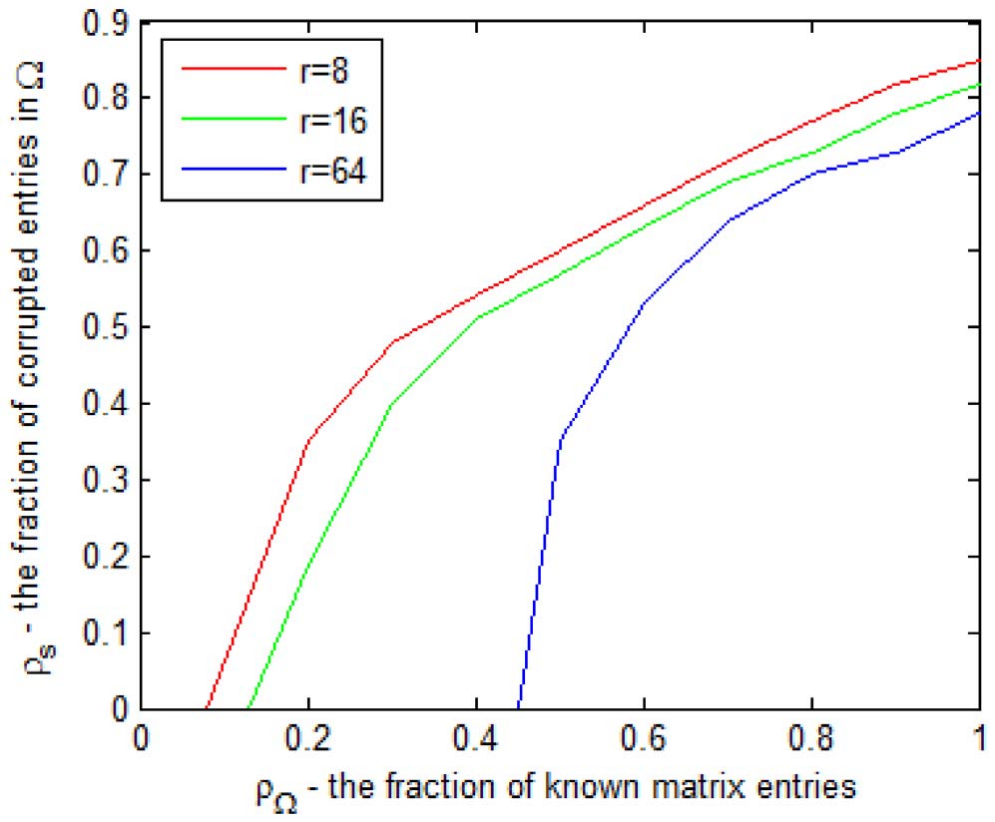
Phase transition with regard to 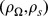
 **of the proposed ALM algorithm.** The curves colored red, green and blue define “phase transition” bounds for the case of 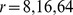, respectively.

As for the experiments of phase transition analysis, we fix 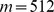, set 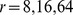 respectively, and vary 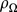 and 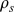 between 0 and 1. For each 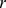 and each 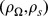 pair, we generate 5 pairs 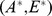 to obtain 5 observed matrices as described above. We deem the recovery successful if the recovered 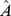 satisfies 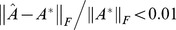 for all the 5 observed data matrices. The curves colored red, green and blue in Figure 1 define “phase transition” bounds for the case of 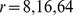, respectively. In Figure 1, the horizontal coordinate indicates 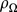, while the vertical coordinate denotes 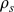. At the points of these curves, all 5 trials were accomplished with success, whereas for the points above the curves, at least one attempt failed. It is assumed that the regions under the curves are “regions of success”. Our experiments show that for fixed 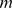, the smaller rank 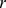 is, the larger area the region of success will have. In fact, when other factors keep unchanged, the recovery performance of the algorithm is inversely proportional to 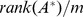. In addition, we can also observe that erasure rate and error probability keep the relationship of interacting.

## Our Denoising Scheme for Mixed Gaussian-Impulse Noise

Image denoising is a research hotspot in the area of image processing all the way. However, in real world, images are typically contaminated by more than one type of noise during image acquisition and transmission process. As a matter of fact, we often encounter the case where an image is corrupted by both Gaussian and impulse noise. Such mixed noise could occur when an image that has already been stained by Gaussian noise in the procedure of image acquisition with faulty equipment suffers impulsive corruption during its transmission over noisy channels successively.

As we know, there usually exist many regularly geometrical textures and similar structures in both natural image and Remote Sensing image, due to the self-similarity and redundancy of image, which makes the grayscale matrix of the image possess low-rank features. Matrix completion can accurately reconstruct low-rank information by exploiting reliable pixels after detecting the isolated noises, which is quite suitable for impulse noise (especially for salt-pepper noise) removal. Robust PCA can recover low-rank matrix from observed matrix with sparse errors, which may deal with the problem of Gaussian noise removal to some extent, since for Gaussian noise, corruptions with large magnitude are rarely distributed while most of the corruptions are located near zero. Luckily, the proposed ALM algorithm for the Robust MC problem, also supplies a powerful technique for removing mixed Gaussian-impulse noise from images with low-rank features.

In this paper, we adopt the two-phase scheme for mixed noise removal: By detecting impulse noises in a noisy image and treating the impulse free entries of the image matrix as available samplings first, and then regarding the Gaussian noises underlying the samplings as arbitrary errors, we can exploit the proposed algorithm to remove mixed noises and to recover the grayscale matrix of the image with low-rank or approximately low-rank features. It should be noted that, we cannot simply use MC for removing impulse noises after detecting them, since the remaining Gaussian noises no longer make the image possess low-rank features; in addition, neither can we directly employ Robust PCA to restore the noisy image at first, because impulse noises destroy the sparsity of large errors, which may greatly decrease the accuracy of the restoration.

The success of two-phase approaches for noise removal relies on the accurate detection of impulse noise. Many impulse noise detectors are proposed in the literatures, e.g. AMF is used to detect salt-pepper noise, while adaptive center-weighted median filter (ACWMF) and rank-ordered logarithmic difference (ROLD) [32] are utilized to detect random-valued impulse noise. In this paper, study on detectors for random-valued impulse noise is beyond the scope of the topic.

For salt-pepper noise, we employ the following strategy to detect it after considering its features taken on in the image: (1) Grayscale thresholding. Specifically, set a fixed thresholding 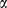, and regard those pixels whose gray-levels are distributed in the interval 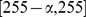 or 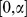 as candidate noises. (2) Median detecting. For current candidate noise, we consider the absolute difference between its gray-level and the median gray-level of its neighborhood pixels. If the absolute difference is larger than another given thresholding 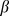, we then treat current candidate noise as true noise. Empirically, when the density of salt-pepper noise namely noise level is high, the window size of the neighborhood for median detecting should be large.

## Experiments and Discussion

This section is devoted to the experimental analysis and discussion of the denoising scheme introduced in the previous section. We compare our ALM algorithm with some recently proposed approaches [12], [15], [16] for dealing with the mixture of impulse and Gaussian noise. Comparison results are presented both under the form of statistical index tables and that of visual effects. To evaluate the quality of the restoration results, peak signal to noise ratio (PSNR) and structural similarity (SSIM) are employed for objective evaluation and for subjective visual evaluation, respectively.

Given an image 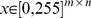, the PSNR of the restored image 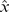 is defined as follows:
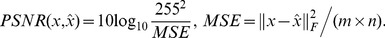
(13)


The larger the value of PSNR is, the better the quality of restoration will be. We can formulate the SSIM between original image and the recovered image as
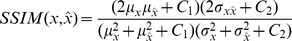
(14)(see [33] for more details). The dynamic range of SSIM is 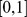, which means a better recovery performance with the SSIM closer to the value 1.

**Figure 2:**
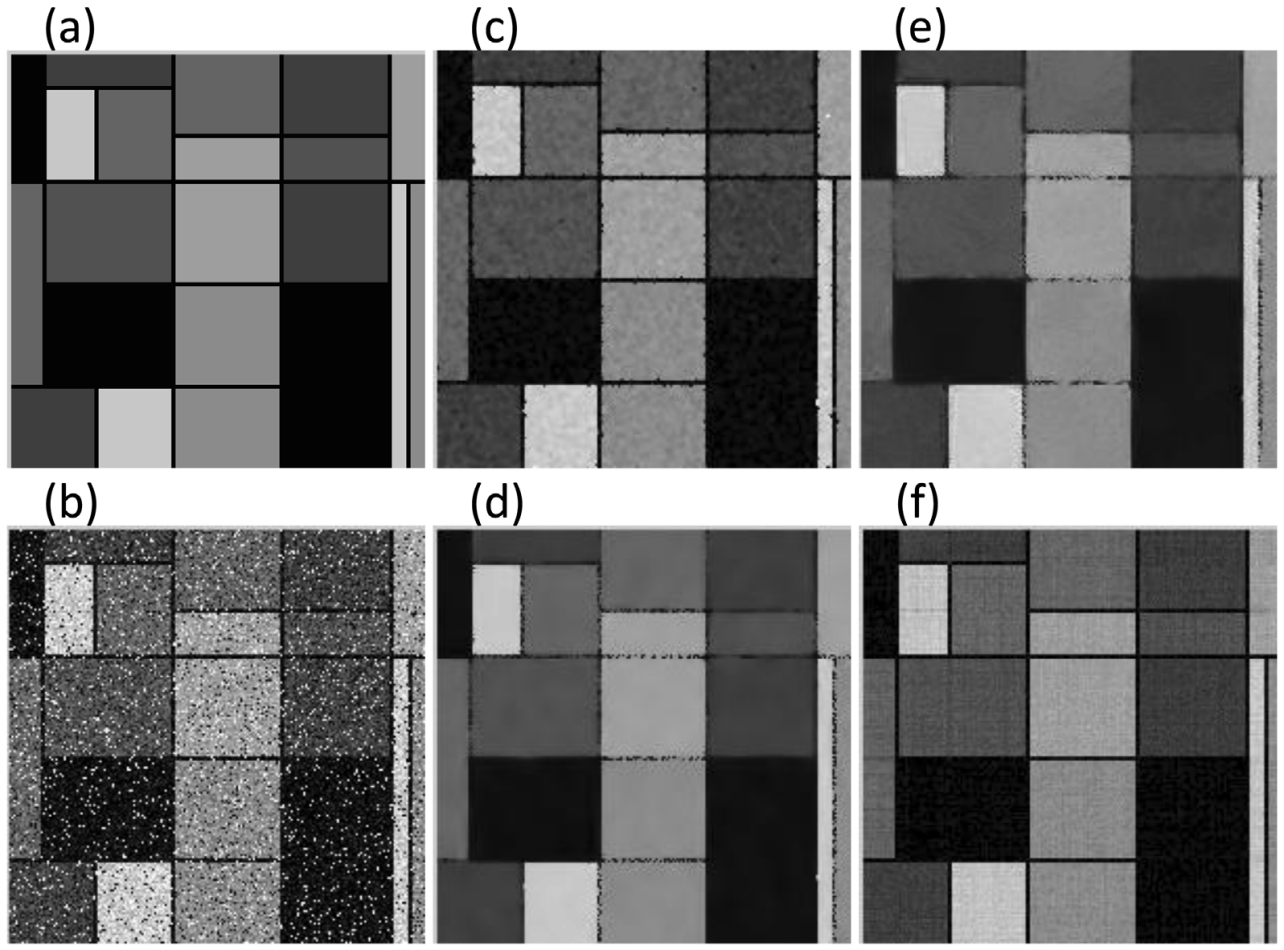
Comparative results on Mondrian image with 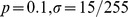
 **.** The PSNR (dB) and SSIM for (b) ∼ (f) are (13.681, 0.883), (27.704, 0.981), (23.542, 0.966), (23.376, 0.966), (**30.158, 0.985**), respectively. Figure (a): Original Image; (b): Noisy Image; (c): Trilateral Filter [12]; (d): Xiao et al. [15]; (e): Xiong and Yin's [16]; (f): Proposed ALM.

As the proposed algorithm is suitable for restoring images with low-rank information, we first choose the classical Mondrian and Barbara image in the field of image processing to do the experiments. In all experiments, parameters of each method have been tuned to yield the best results. On image Mondrian, we successively add Gaussian noise with 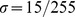 and salt-pepper noise with 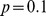. The visual comparison of the four approaches is presented in Figure 2, from where we will see that our method can simultaneously remove Gaussian noise and salt-pepper noise in the restoration phase, and well reconstruct the original image with low-rank structures; above all, it can better preserve details and edges in the image. By contrast, the other three approaches either cannot thoroughly remove the mixed noises or will destroy fine structures. The values of PSNR and SSIM also demonstrate that the ALM-based approach outperforms other methods significantly not only in objective index, but also in subjective visual effect.

**Figure 3:**
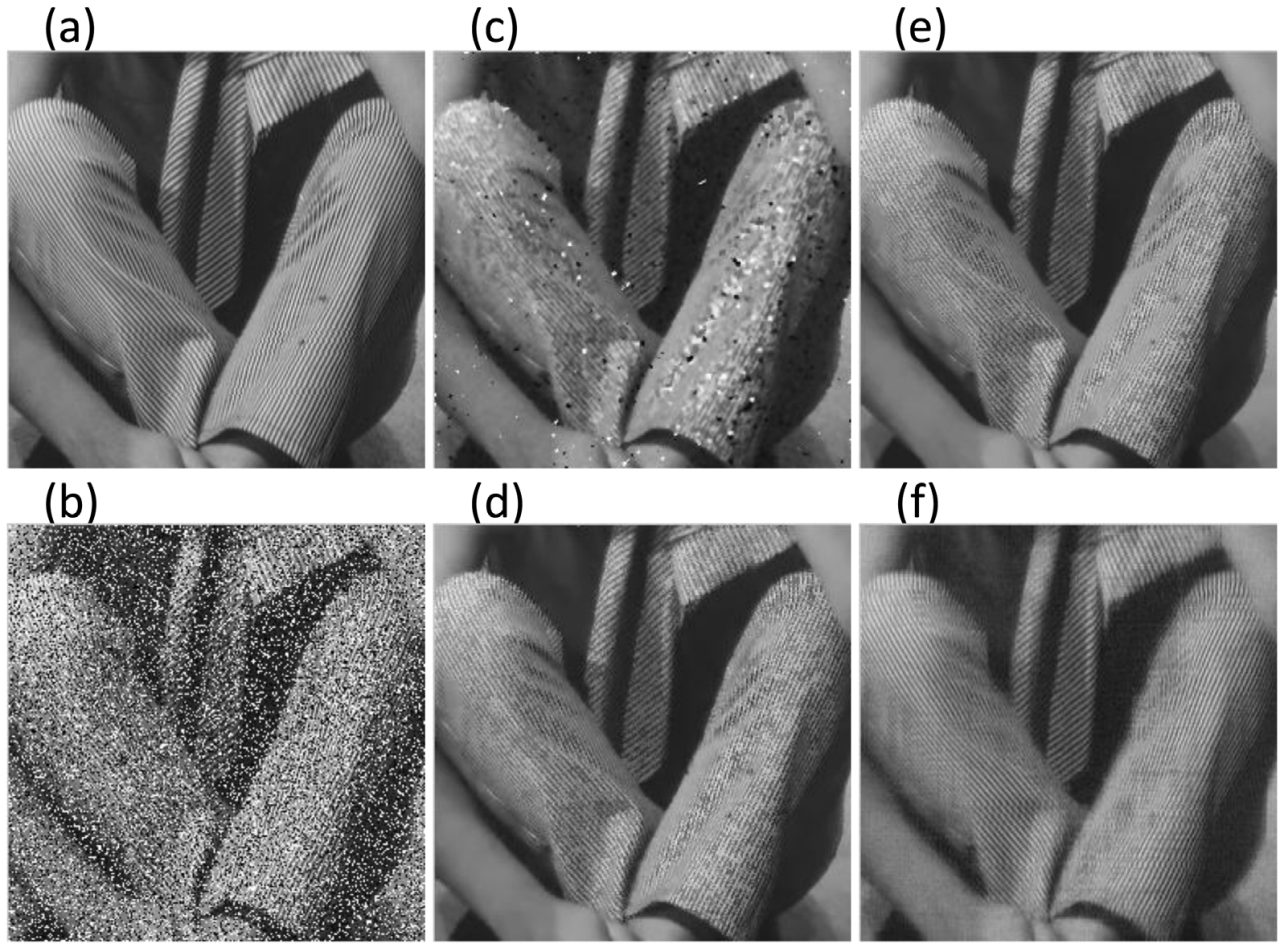
Comparison of visual effect on Barbara image when 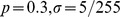
 **.** Figure (a): Original Image; (b): Noisy Image; (c): Trilateral Filter [12]; (d): Xiao et al. [15]; (e): Xiong and Yin's [16]; (f): Proposed ALM.

In the following experiment, image Barbara abundant in geometrical textures, is corrupted by Gaussian noise with zero mean and different standard deviations 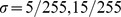 first, and then we add salt-pepper noise with different levels 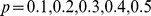 on the image. Comparisons of statistical indices under different combinations of 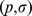 are provided in Table 2, and the comparison of visual effect when 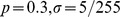 is also shown in Figure 3. From Figure 3, we also observe that our method can preserve regularly geometrical textures perfectly while removing the mixed Gaussian-impulse noises from the image. However, other approaches will more or less blur the textured information consisting in the image. Meanwhile, we can see that the differences of recovery performance between the proposed ALM algorithm and the other three methods will be more remarkable when the level of salt-pepper noise is relatively high while the variance of Gaussian noise is small.

**Table 2:** Comparisons of statistical indices under different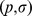 on Barbara Image.

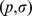	Statistical Index	Noisy Image	ROAD-Trilateral [12]	Xiao et al. [15]	Xiong and Yin's [16]	Proposed ALM
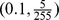	PSNR(dB)	15.132	21.528	27.027	26.472	**27.163**
	SSIM	0.956	0.962	0.983	0.984	**0.986**
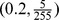	PSNR(dB)	12.184	19.712	26.325	25.713	**26.386**
	SSIM	0.913	0.949	0.980	0.981	**0.982**
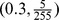	PSNR(dB)	10.407	17.652	25.217	24.688	**25.287**
	SSIM	0.870	0.933	0.975	0.976	**0.977**
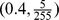	PSNR(dB)	9.179	14.854	24.017	23.438	**24.237**
	SSIM	0.827	0.906	0.969	0.970	**0.971**
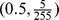	PSNR(dB)	8.223	12.096	22.977	22.289	**23.315**
	SSIM	0.785	0.857	0.964	0.962	**0.965**
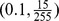	PSNR(dB)	14.910	20.726	**26.041**	25.892	25.385
	SSIM	0.938	0.954	**0.978**	0.978	0.975
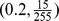	PSNR(dB)	12.044	19.432	**25.542**	25.280	25.067
	SSIM	0.896	0.945	0.975	0.976	0.973
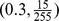	PSNR(dB)	10.349	16.838	**24.574**	24.256	24.182
	SSIM	0.855	0.928	**0.971**	0.971	0.970
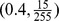	PSNR(dB)	9.131	13.945	**23.555**	23.076	23.307
	SSIM	0.814	0.893	0.965	0.965	**0.965**
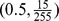	PSNR(dB)	8.206	11.604	22.492	21.955	**22.684**
	SSIM	0.776	0.846	0.961	0.958	**0.962**

**Figure 4:**
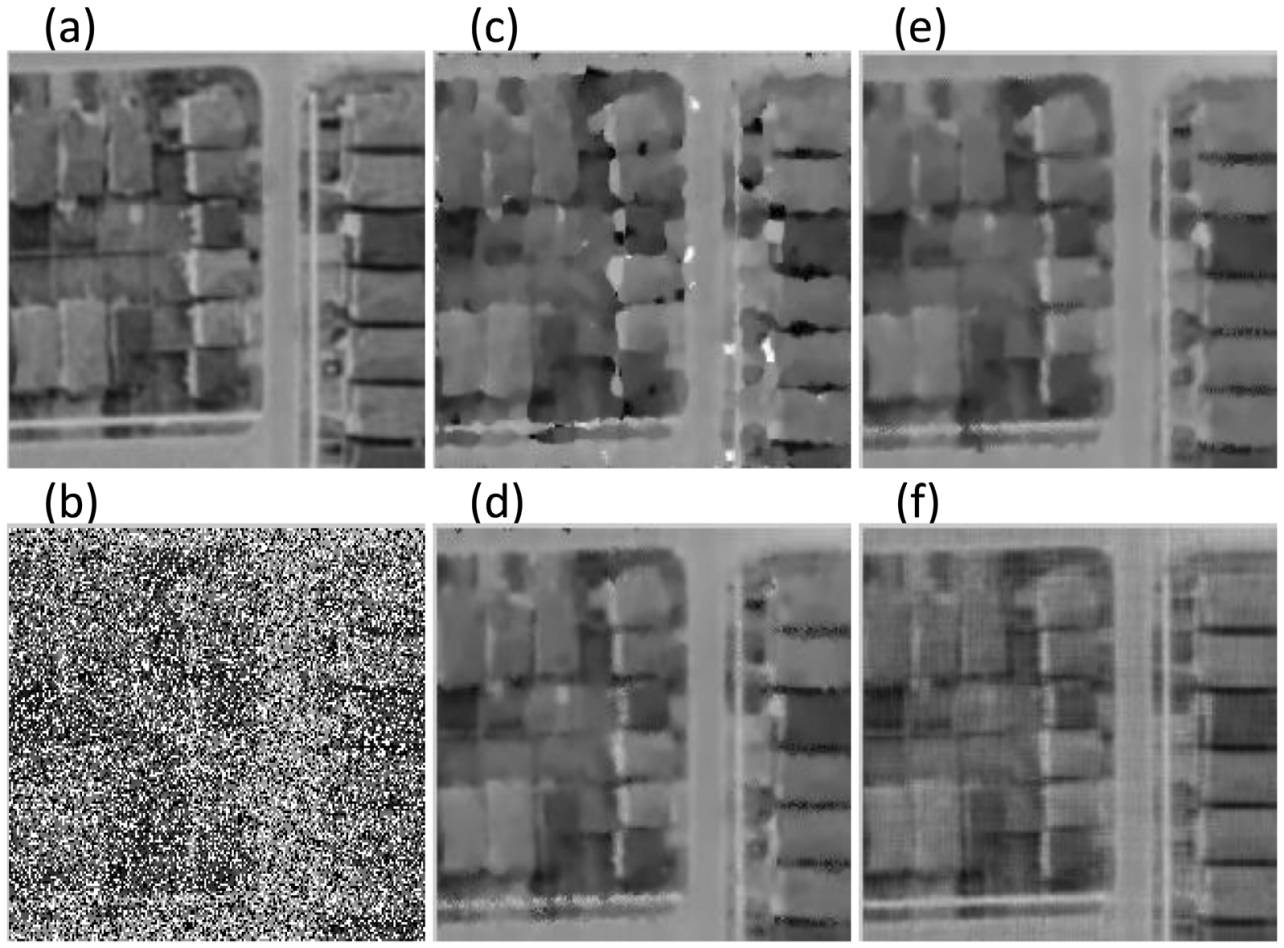
Comparison of visual effect on RS image when 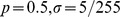
 **.** Figure (a): Original Image; (b): Noisy Image; (c): Trilateral Filter [12]; (d): Xiao et al. [15]; (e): Xiong and Yin's [16]; (f): Proposed ALM.

To better demonstrate the excellent performance of our method, we do a further comparison experiment of the four approaches in removing mixed Gaussian-impulse noise from Remote Sensing image with low-rank features. As in the previous experiment, we add Gaussian and salt-pepper noise with different values of 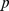 and 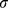 on RS image. The experimental results are presented in Figure 4 and Table 3. Again, it shows that our method can better remove the mixed noise and restore the image especially when the level of impulse noise is relatively high while Gaussian noise is small.

**Table 3:** Comparisons of statistical indices under different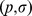 on RS Image.

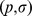	Statistical Index	Noisy Image	ROAD-Trilateral [12]	Xiao et al. [15]	Xiong and Yin's [16]	Proposed ALM
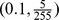	PSNR(dB)	15.620	33.976	34.459	36.079	**36.246**
	SSIM	0.954	0.994	0.994	0.996	**0.996**
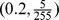	PSNR(dB)	12.509	30.145	33.993	34.918	**35.170**
	SSIM	0.910	0.989	0.993	0.995	**0.995**
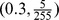	PSNR(dB)	10.691	27.797	32.550	33.037	**33.691**
	SSIM	0.861	0.983	0.992	0.992	**0.993**
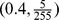	PSNR(dB)	9.559	24.160	30.941	31.603	**32.317**
	SSIM	0.828	0.976	0.988	0.990	**0.991**
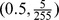	PSNR(dB)	8.590	19.342	29.151	29.097	**30.892**
	SSIM	0.787	0.948	0.984	0.985	**0.988**
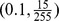	PSNR(dB)	15.144	29.618	29.893	**31.581**	28.385
	SSIM	0.932	0.985	0.986	**0.989**	0.981
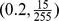	PSNR(dB)	12.393	27.657	29.809	**30.978**	27.943
	SSIM	0.892	0.981	0.985	**0.988**	0.979
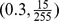	PSNR(dB)	10.739	26.382	29.277	**30.168**	27.481
	SSIM	0.851	0.976	0.984	**0.987**	0.977
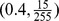	PSNR(dB)	9.563	23.601	28.385	**29.103**	26.995
	SSIM	0.829	0.969	0.980	**0.985**	0.975
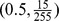	PSNR(dB)	8.543	16.873	26.993	**27.606**	26.617
	SSIM	0.774	0.937	0.975	**0.977**	0.974

**Figure 5:**
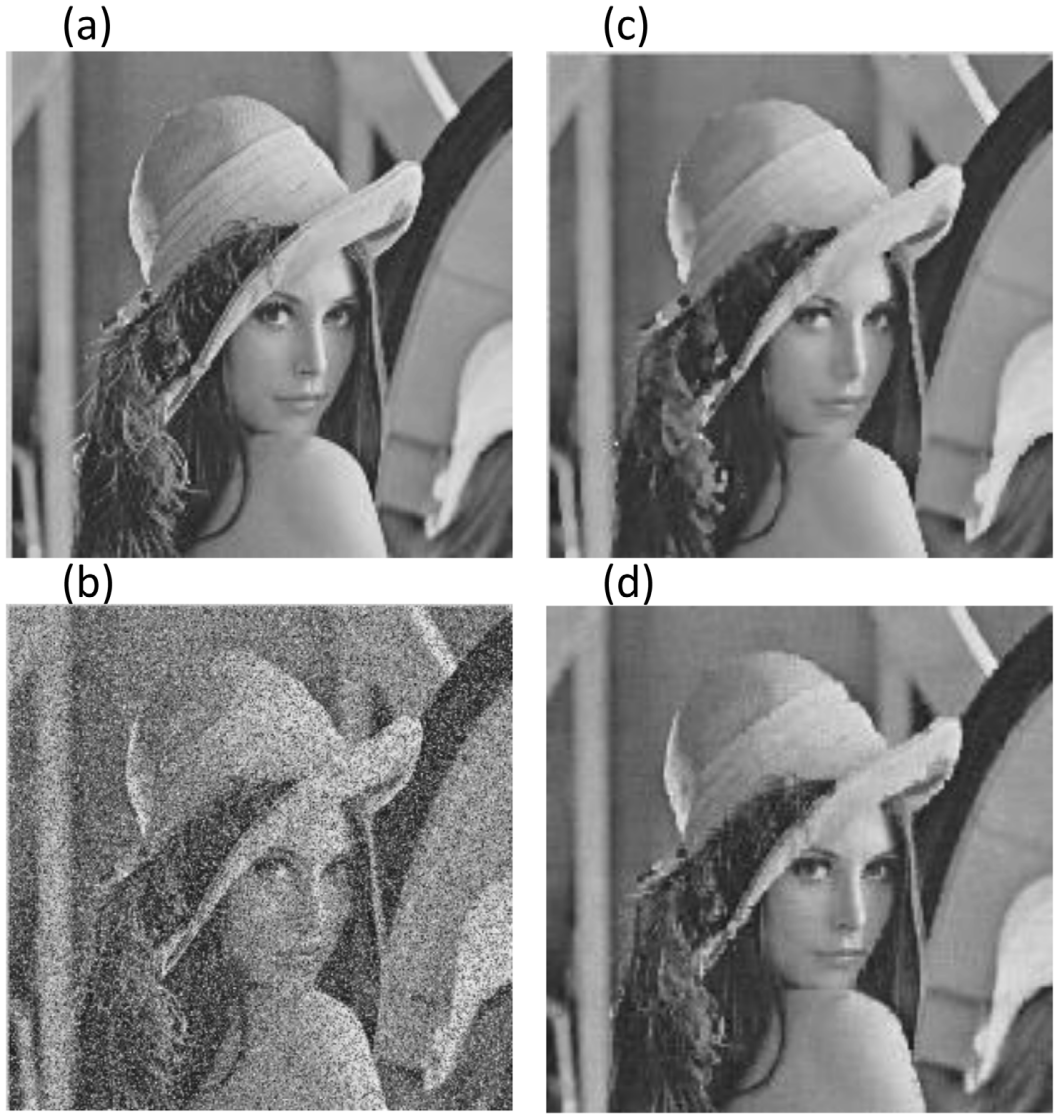
Comparative results on Lena image with 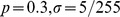
 **.** The PSNR (dB) and SSIM for (b) ∼ (d) are (10.669, 0.887), (27.237, 0.987), **(28.070, 0.989)**, respectively. Figure (a): Original Lena; (b): Noisy Lena; (c): Trilateral Filter; (d): Proposed ALM.

**Figure 6:**
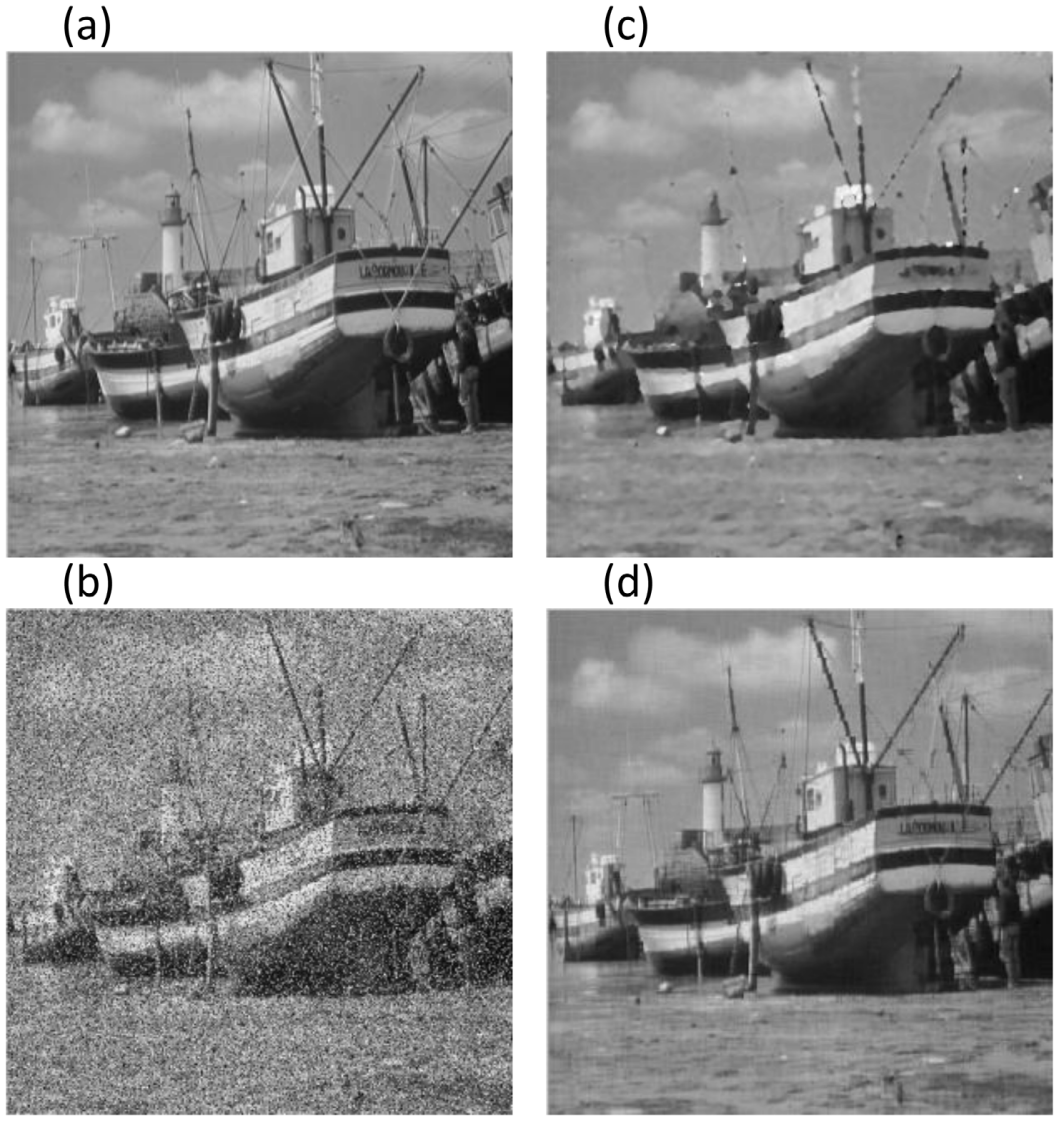
Comparative results on Boat image with 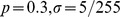
 **.** The PSNR (dB) and SSIM for (b) ∼ (d) are (10.696, 0.909), (23.737, 0.973), **(27.192, 0.985)**, respectively. Figure (a): Original Boat; (b): Noisy Boat; (c): Trilateral Filter; (d): Proposed ALM.

The above experiments mainly involve images with significant low-rank features such as similar structures and regular textures, which violates the fact that most of images in the real world cannot possess globally low-rank features. However, most local parts in the image will meet low-rank or approximately low-rank condition, due to the self-similarity and spatial correlation of images. Consequently, we can utilize the proposed algorithm for image de-noising via block processing-based technique. The following experiments demonstrate the results of removing mixed Gaussian-impulse noise from Lena and Boat images, compared to trilateral filter method. Both of the noisy images are of size 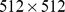 pixels, either divided into many image blocks of size 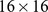 pixels. The de-noising results for Lena and Boat are presented in Figure 5 and Figure 6 respectively, which also show the better performance of our method over trilateral filter. We can see that the details of hairs are not preserved well in Figure 5c, while in Figure 6c, some structures of the masts on the boats are lost.

## Conclusion

In this paper, regarding the problem of low-rank matrix recovery from incomplete and corrupted samplings of its entries namely the Robust MC problem, we construct a mathematical model based on convex optimization, and put forward a novel and effective ALM algorithm, to solve this kind of optimization problem which can be considered as the extension of the Robust PCA and the MC problem. Experiments on performance analysis and mixed Gaussian-impulse noise removal demonstrate the reliability and practicability of the proposed algorithm, which also show that our method can well preserve details and textures and keep the consistency of structures, while simultaneously removing mixed noises from images with low-rank features. By virtue of the novelty and powerful advantages, the approach will bring promising application value in the fields of data mining, image processing, machine learning, RS information processing and so forth.
